# Emerging Role of Emergency Physicians in Antimicrobial Stewardship

**DOI:** 10.7759/cureus.93058

**Published:** 2025-09-23

**Authors:** Hiroaki Taniguchi

**Affiliations:** 1 Department of Emergency Medicine, Japan Self Defense Forces Sapporo Hospital, Sapporo, JPN

**Keywords:** antibiotic resistance, antimicrobial stewardship, emergency department, emergency physicians, prescribing appropriateness

## Abstract

The emergency department (ED) is increasingly recognized as a critical site for antimicrobial stewardship, where most initial antibiotic decisions occur, and emergency physicians (EPs), previously regarded as primarily focused on empiric treatment, are now emerging as leaders. Policy milestones, including accreditation standards and national guidelines, have catalyzed this transition, yet physician roles remain heterogeneous, and evidence on long-term outcomes is limited. Drawing on English-language literature, including intervention studies, descriptive reports, and policy documents, this editorial highlights how EPs contribute through guideline development, audit and feedback, culture follow-up, formulary oversight, rapid diagnostics, and educational initiatives. Evidence consistently shows improvements in prescribing appropriateness, reduced use of broad-spectrum agents, and faster optimization without compromising safety. However, barriers such as time pressure, professional culture, role ambiguity, and limited resources remain persistent. Importantly, the leadership of EPs has proven central in overcoming these obstacles, especially when supported by institutional commitment and integrated workflows. Their engagement is not only improving immediate prescribing practices but also represents a critical frontline defense against antimicrobial resistance and multidrug-resistant organisms. Looking forward, formalizing physician leadership, embedding stewardship in emergency medicine training, and building ED-specific data systems are essential to sustain safe and effective antibiotic use.

## Editorial

The emergency department (ED) is a frontline setting where most initial antimicrobial prescribing decisions are made, making it a critical site for antimicrobial stewardship (ASP) and for addressing the growing threat of antimicrobial resistance (AMR) and multidrug-resistant organisms (MDROs) [1,2]. Historically, emergency physicians (EPs) were regarded as clinicians whose primary responsibility was the rapid initiation of empiric treatment and referral, reflecting the urgency of emergency care [1,3]. Over the past decade, however, their role has expanded to include active participation and leadership. This shift has been supported by major policy milestones, including national stewardship guidelines and accreditation standards that formally recognized the ED as a stewardship site [4]. Despite these advances, EP involvement remains heterogeneous and unstandardized across institutions, and evidence on long-term patient outcomes and program sustainability is limited [5]. Moreover, relatively few systematic reviews or high-quality studies have specifically examined EP-led stewardship, highlighting a gap in the literature and underscoring the need for further synthesis [6,7]. This editorial highlights how EPs are increasingly positioned as champions of ASP, reviews key examples of their contributions, and considers the opportunities and challenges that will shape their role in the years ahead. The discussion is structured around four recurring themes in the literature: the scope of EP involvement, specific physician-led interventions, reported outcomes, and the barriers and facilitators shaping implementation. The emphasis is on clinical relevance and practical implications for emergency medicine practice and policy.

Scope of EP involvement

Historically, ASP efforts concentrated on inpatient units, leaving the ED underrepresented [1,3]. Recent policy changes, such as accreditation requirements for hospital ASPs, have prompted explicit inclusion of EDs. In response, EPs have increasingly taken on leadership roles as champions and liaisons in ED-focused stewardship programs, particularly relevant given rising concerns about AMR and MDROs and findings from a national survey that many EDs lack regular AMS training, surveillance of antibiotic use, and ED-level AMR data [2,8,9]. The American College of Emergency Physicians 2020 policy statement explicitly “supports and encourages the engagement of emergency physicians and EDs in antimicrobial stewardship efforts” [4]. In practice, this often entails appointing an EP champion who collaborates with hospital ASP leadership and coordinates stewardship activities among ED staff [8,10].

Within multidisciplinary ASP teams, EPs typically co-lead alongside pharmacists and infectious disease specialists. The EP champion frequently serves as a bridge between the hospital ASP committee and frontline clinicians, facilitating implementation of guidelines, promoting adherence to new protocols or order sets, and providing feedback to colleagues. A recent review identified the establishment of physician-pharmacist teams as a “crucial first step” in operationalizing stewardship interventions that fit the unique workflow of emergency care [9]. Pharmacist-EP collaboration has been especially emphasized in studies evaluating antibiotic selection, dosing, and culture result follow-up.

Beyond the ED, EPs have extended stewardship activities into urgent care and outpatient emergency settings by piloting antibiotic prescribing guidelines. In rural or resource-limited hospitals, EP champions have adapted stewardship protocols to local realities where ID specialists may be scarce. In outbreak scenarios, such as Ebola and COVID-19, EPs have taken on stewardship-related leadership by ensuring judicious antimicrobial use while implementing infection control measures under pressure [1,9].

EP-led ASP interventions

EPs have led or co-led a diverse range of ASP initiatives in the ED. These interventions aim to address the unique challenges of acute care, such as high patient turnover, time pressure, and limited diagnostic certainty, while improving prescribing quality without delaying urgent treatment. Condition-specific guidelines and care pathways, particularly for pneumonia, sepsis, and urinary tract infections, have been developed with EP input and shown to reduce inappropriate prescribing [1,8]. Similarly, formulary restrictions and electronic health record order sets - often co-designed by EP leaders - have helped curb unnecessary broad-spectrum antibiotic use while preserving rapid access for critically ill patients, thereby reducing selective pressures that drive AMR and MDRO emergence. These interventions are more likely to succeed when aligned with the ED workflow, ensuring they do not delay time-sensitive care.

In addition to protocols, review mechanisms are an important stewardship tool. Although direct ED-based evidence remains limited, reviews note that audit-and-feedback strategies involving EPs and pharmacists could support early discontinuation of unnecessary antibiotics once cultures return negative [8]. A more consistently reported intervention is post-discharge culture follow-up. Programs where EPs and pharmacists jointly review results and contact patients for therapy adjustments have reduced infection-related return visits and saved substantial physician time, highlighting the value of physician-pharmacist collaboration [1].

EPs have also pioneered rapid diagnostic strategies. The use of multiplex PCR panels and procalcitonin assays has been shown to guide targeted therapy and reduce unnecessary antibiotic initiation, an approach critical for slowing AMR spread in acute care settings. In a study evaluating multiplex respiratory pathogen testing with targeted educational intervention, prescribing practices in the ED improved significantly [11]. Recent guidelines further endorse these approaches, underscoring the role of EPs in their implementation [10]. Education and behavioral nudges are central to changing prescribing culture. EPs have organized clinician training, distributed prescribing feedback reports, and implemented stewardship campaigns. For instance, one study described local adaptation of the MITIGATE toolkit across urgent care centers, primary clinics, and an ED, highlighting how EP leadership can effectively shape ASP culture and contribute to global AMR mitigation efforts [12]. Although the effect sizes were modest, the study illustrates how EP leadership can effectively shape ASP culture within acute care environments.

Outcomes of EP involvement in ASP

Evidence on outcomes of EP-led stewardship in the ED is limited but shows consistent benefits. Process outcomes are most robust: EP-driven guidelines for pneumonia, sepsis, and urinary tract infections improve prescribing appropriateness [1,8], which in turn contributes to reducing selective pressure and slowing the spread of AMR and MDROs [2,10]. Post-discharge culture follow-up, often co-managed with pharmacists, reduces infection-related return visits and optimizes therapy, while audit-and-feedback efforts remain less common but promising. Although direct evidence linking ED stewardship interventions to post-hospitalization MDRO incidence is scarce, prior reviews emphasize that early prescribing decisions in the ED disproportionately shape inpatient antibiotic exposure, suggesting that strengthening these practices may help mitigate downstream MDRO risks [1,8].

Clinical outcomes are less conclusive. Mortality benefits are rarely shown, though some studies suggest fewer adverse drug events and revisit rates [5]. A controlled sepsis protocol reported a survival benefit, but confirmation in larger cohorts is required [13].

Program sustainability depends on local leadership. Multi-component initiatives anchored by EP champions have reduced antibiotic use, costs, and *Clostridioides difficile* infection incidence without raising mortality, supporting feasibility in ED settings [6]. Surveys indicate EDs with formal stewardship champions maintain more consistent practices. Finally, meta-analytic data reinforce multidisciplinary value: pharmacist-led stewardship, typically in collaboration with EPs, was associated with nearly threefold higher odds of appropriate prescribing and 18.9 hours faster optimization, without safety trade-offs [7].

Barriers and facilitators to EP engagement in ASP

Implementing ASP in the ED poses distinct challenges. Time pressure and high patient volume limit opportunities for reflection, making stewardship tasks such as antibiotic documentation or justification difficult during busy shifts [8]. Cultural barriers also exist: the ED tradition of rapid, autonomous decision-making can create resistance to restrictions or peer review until reframed as patient safety practices [5]. Structural issues, including role ambiguity between infectious disease specialists, pharmacists, and ED leadership, rotational staffing, and lack of ED-specific prescribing data, further hinder sustainability [9]. Limited pharmacy support and IT infrastructure add to these constraints [1]. These barriers are particularly concerning in the context of AMR and the rising prevalence of MDROs, where inappropriate prescribing in the ED may accelerate resistance development [2].

Counterbalancing these barriers, several facilitators have been highlighted. Visible EP champions with institutional backing are repeatedly cited as decisive in launching and sustaining programs [6]. Multidisciplinary engagement of pharmacists, nurses, and infection prevention staff, supported by targeted education and peer comparison, fosters shared accountability [1]. Workflow integration-such as embedding stewardship prompts in electronic health records, aligning sepsis order sets with guidelines, and ensuring access to rapid diagnostics-reduces friction and promotes adoption [9]. Finally, feedback loops and external toolkits like MITIGATE and the 2024 European Society of Clinical Microbiology and Infectious Diseases guidelines provide structured frameworks that reinforce ongoing adherence [10,12].

Looking ahead

EPs are increasingly recognized not only as frontline prescribers but also as leaders capable of shaping stewardship culture in ED. While earlier reviews have focused on ED stewardship interventions in general, much less attention has been given to the unique professional role of EPs themselves [5,8]. Highlighting this shift from passive participation to active leadership underscores the value of physicians who understand the pace and culture of emergency care.

Future progress depends on embedding stewardship into daily ED practice. Priorities include appointing EP champions with institutional support, integrating decision support and feedback systems, extending stewardship into emergency medical services and public health, and ensuring its inclusion in emergency medicine training. These measures are particularly critical in the context of AMR and the spread of MDROs, where early prescribing decisions in the ED can have disproportionate downstream effects [2, 10].

The key challenge is cultural: balancing the autonomy and speed of emergency medicine with the accountability of stewardship. With visible physician leadership and multidisciplinary collaboration, stewardship can strengthen rather than hinder emergency care, positioning EPs as central actors in the global fight against AMR and MDROs.

## References

[1] Pulia M, Redwood R, May L (2018). Antimicrobial stewardship in the emergency department. Emerg Med Clin North Am.

[2] Pin M, Somasundaram R, Wrede C, Schwab F, Gastmeier P, Hansen S (2022). Antimicrobial resistance control in the emergency department: a need for concrete improvement. Antimicrob Resist Infect Control.

[3] Trinh TD, Klinker KP (2015). Antimicrobial stewardship in the emergency department. Infect Dis Ther.

[4] (2020). Antimicrobial stewardship. Ann Emerg Med.

[5] Losier M, Ramsey TD, Wilby KJ, Black EK (2017). A systematic review of antimicrobial stewardship interventions in the emergency department. Ann Pharmacother.

[6] Savoldi A, Foschi F, Kreth F (2020). Impact of implementing a non-restrictive antibiotic stewardship program in an emergency department: a four-year quasi-experimental prospective study. Sci Rep.

[7] Kooda K, Canterbury E, Bellolio F (2022). Impact of pharmacist-led antimicrobial stewardship on appropriate antibiotic prescribing in the emergency department: a systematic review and meta-analysis. Ann Emerg Med.

[8] May L, Martín Quirós A, Ten Oever J, Hoogerwerf J, Schoffelen T, Schouten J (2021). Antimicrobial stewardship in the emergency department: characteristics and evidence for effectiveness of interventions. Clin Microbiol Infect.

[9] Ruiz-Ramos J, Escolà-Vergé L, Monje-López ÁE, Herrera-Mateo S, Rivera A (2023). The interventions and challenges of antimicrobial stewardship in the emergency department. Antibiotics (Basel).

[10] Schoffelen T, Papan C, Carrara E (2024). European society of clinical microbiology and infectious diseases guidelines for antimicrobial stewardship in emergency departments (endorsed by European association of hospital pharmacists). Clin Microbiol Infect.

[11] Durant TJ, Kubilay NZ, Reynolds J, Tarabar AF, Dembry LM, Peaper DR (2020). Antimicrobial stewardship optimization in the emergency department: the effect of multiplex respiratory pathogen testing and targeted educational intervention. J Appl Lab Med.

[12] Huang J, Kassamali Escobar Z, Bouchard TS (2021). Finding the path of least resistance: locally adapting the MITIGATE toolkit in emergency departments and urgent care centers. Infect Control Hosp Epidemiol.

[13] Peltan ID, Bledsoe JR, Jacobs JR (2024). Effectiveness and safety of an emergency department code sepsis protocol: a pragmatic clinical trial. Ann Am Thorac Soc.

